# Asterias: A Parallelized Web-based Suite for the Analysis of Expression and aCGH Data

**Published:** 2007-02-03

**Authors:** Andreu Alibés, Edward R. Morrissey, Andrés Cañada, Oscar M. Rueda, David Casado, Patricio Yankilevich, Ramón Díaz-Uriarte

**Affiliations:** Statistical Computing Team, Structural and Computational Biology Programme, Spanish National Cancer Center (CNIO), Melchor Fernández Almagro 3, Madrid, 28029, Spain

**Keywords:** microarray, aCGH, classification, prediction, parallel computing, web-based application

## Abstract

The analysis of expression and CGH arrays plays a central role in the study of complex diseases, especially cancer, including finding markers for early diagnosis and prognosis, choosing an optimal therapy, or increasing our understanding of cancer development and metastasis. Asterias (http://www.asterias.info) is an integrated collection of freely-accessible web tools for the analysis of gene expression and aCGH data. Most of the tools use parallel computing (via MPI) and run on a server with 60 CPUs for computation; compared to a desktop or server-based but not parallelized application, parallelization provides speed ups of factors up to 50. Most of our applications allow the user to obtain additional information for user-selected genes (chromosomal location, PubMed ids, Gene Ontology terms, etc.) by using clickable links in tables and/or figures. Our tools include: normalization of expression and aCGH data (DNMAD); converting between different types of gene/clone and protein identifiers (IDconverter/IDClight); filtering and imputation (preP); finding differentially expressed genes related to patient class and survival data (Pomelo II); searching for models of class prediction (Tnasas); using random forests to search for minimal models for class prediction or for large subsets of genes with predictive capacity (GeneSrF); searching for molecular signatures and predictive genes with survival data (SignS); detecting regions of genomic DNA gain or loss (ADaCGH). The capability to send results between different applications, access to additional functional information, and parallelized computation make our suite unique and exploit features only available to web-based applications.

## Introduction

Gene expression data from DNA microarrays have had a central role in the study of complex diseases, especially cancer and, in the last 10 years, hundreds of papers using gene expression data from microarray studies of cancer patients have been published (Rhodes and Chinnaiyan, 2005). However, the use of data from microarray studies for early diagnosis and prognosis and to help to choose an optimal therapy, or to increase our understanding of cancer development and metastasis, faces several challenges (Rhodes and Chinnaiyan, 2005; Michiels et al. 2005; Ransoho, 2005). Some of the most relevant challenges are the validation of the robustness and stability of analysis’ results, the biological interpretation of those results, and the integration of information from other sources (e.g. functional annotation). To approach these difficulties we need computationally efficient applications that can analyze massive amounts of data, and that make no compromises with the statistical rigor of the analysis.

A large number of web applications for genomic data are available but many have been developed for a single task (e.g. GEMS—Wu and Kasif, 2005, for biclustering; VAMPIRE—Hsiao et al. 2005, for differential gene expression analysis, CAPweb—Liva et al. 2006, for aCGH analysis). From a user’s point of view, integrated suites can be much more appealing: they show a common, unified interface, similar requirements regarding data formatting, and allow the user to perform complete sets of analysis (e.g. starting from data normalization, following with data merging by gene ID, and finishing with the search of differentially expressed genes and class prediction models). Some suites include RACE (Psarros et al. 2005), MIDAW (Romualdi et al. 2005), Gepas (Montaner et al. 2006; Vaquerizas et al. 2005; Herrero et al. 2004; 2003), and CARMAweb (Rainer et al. 2006).

Our suite Asterias, as some of the other available suites, offers integrated analysis, the possibility to use either the full suite or just specific applications, and access to additional functional information. Asterias’ unique features are: a) Asterias is explicitly designed to take advantage of web-based applications running on a multi-server site, by using load-balancing and, more importantly, parallelized execution. Parallel computing (Pacheco, 1997; Foster, 1995) can result in dramatic decreases in the time a user must wait to obtain results (e.g. Pomelo II, the parallelized version of Pomelo—Vaquerizas et al. 2005; Herrero et al. 2004, can achieve speed ups of factors up to 50 in our computing cluster). By itself, this efficient use of a multi-server infrastructure makes Asterias unique. b) Asterias leads to a careful examination of the problem of multiple solutions. Studies about class prediction (e.g. cancer vs. non-cancer) with genomic data have shown repeatedly (Michiels et al. 2005; Díaz-Uriarte 2005; Díaz-Uriarte and Alvarez de Andres, 2006; Ein-Dor et al. 2005; Somorjai et al. 2003; Pan et al. 2005; Yeung and Bumgarner, 2003) that many problems have multiple “solutions” (sets of genes and models) with equivalent predictive capacity. All the prediction tools in our suite Asterias (Tnasas, SignS, GeneSrF) provide detailed reports about multiple solutions, by using either cross-validation or the bootstrap. c) We emphasize careful testing of our applications and, a unique feature in web-based applications, provide complete source code of our automated testing procedures. d) Asterias integrates tools that cover the whole range of needs of many labs and researchers (normalization, filtering and missing value imputation, differential gene expression, class prediction, survival analysis, and aCGH analysis), being the only suite that incorporates searching for large sets of predictive genes (GeneSrF) and prediction of survival data (SignS).

## Functionality

**Figure 1.**
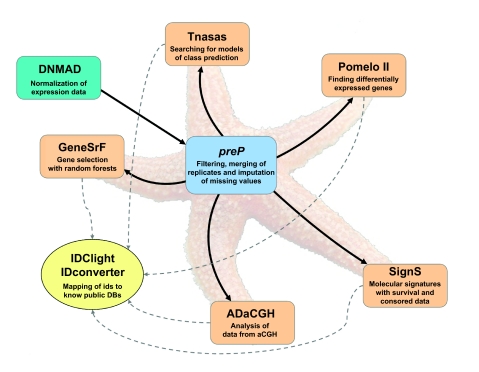
Asterias suite. Relationships between the applications currently available in Asterias. An arrow indicates the possibility of automatically transferring the output from one application (origin of the arrow) as input for another application (end of row). All applications can also be accessed independently. Photo credit: the starfish is a modified image taken from the Wikipedia entry for Asterias (http://en.wikipedia.org/wiki/Image:Asterias_rubens.jpg), and belongs to Hans Hillewaert.

Asterias provides an integrated set of freely-available tools that allows for comprehensive analysis of expression and aCGH data, from normalization to searching for class and survival prediction models and integration of additional functional information. Figure 1 shows the applications and their relationships. All tools are accessible from preP, but can also be accessed directly, and preP can be accessed either directly or from DNMAD. The functionality and analysis provided by each application are:

**DNMAD** Diagnosis and normalization of array data (both expression and aCGH).

- Diagnostic plots to identify possible spatial patterns, arraying problems, and differences in spread among arrays and subarrays.
- Print-tip and global loess-based normalization.
- Use of flags to determine points to exclude and points to normalize but not use for determining the normalization curve.
- Three options for background correction.
- User-specified color ratio (red(Cy5)/green(Cy3) vs. green(Cy3)/red(Cy5)).
- Input as GPR files or custom formatted files, and upload of uncompressed or compressed files.

**preP** Preprocessing of array data.

- Filtering genes with missing data.
- Data imputation using KNN (Troyanskaya et al. 2001).
- Merging of replicate spots in the array.
- Elimination of constant genes/clones/spots.

**IDconverter** Mapping of clone, gene and protein ids to known public databases.

- Output ids: 8 gene ids, 3 clone ids, 5 protein ids, plus PubMed references, GO terms, and KEGG and Reactome pathways. Chromosomal location from two sources.
- Several output formats: HTML, tab separated text file and spreadsheet file.

**IDClight** Same as IDconverter, but input coming-directly from URL.

**Pomelo II** Finding differentially expressed genes.

- For differential expression associated to class differences (using t-test, paired t-test, or ANOVA), a continuous variable (linear regression), or survival time (via Cox models).
- Unadjusted and FDR-adjusted p-values.
- For t-test, ANOVA, and regression, p-values can be obtained by data permutation.
- Empirical bayes moderated statistics for t-test and ANOVA.
- Addition of clinical covariates in linear models.
- Heatmaps with gene dendrograms of user-selected subsets of genes (filtering by statistic, absolute value of statistic, p-value and adjusted p-value and number of genes).

**Tnasas** Searching for models of class prediction.

- Five different class-prediction algorithms (support vector machines, nearest neighbor, discriminant analysis, random forest, and shrunken centroids).
- Three different gene ranking methods (between-to-within sums of squares—F-ratio—, Wilcoxon statistic, random forest).
- Honest assessment of prediction error rate using double cross-validation.
- Assessment of the relationship between number of genes in class prediction models and error rate.
- Comprehensive analysis of stability of solutions for both “best” number of genes and identity of selected genes.

**GeneSrF** Gene selection for classification problems using random forest. Targeted towards identifying both small, non-redundant sets of genes with good predictive performance (as explained in Díaz-Uriarte and Alvarez de Andres, 2006) as well as large sets of genes (including redundant genes) related to the outcome of interest.

- Honest assessment of prediction error rate using the bootstrap.
- Assessment of the relationship between number of genes in class prediction models and error rate.
- Comprehensive analysis of stability of solutions for both “best” number of genes and identity of selected genes and selection probability plots.
- Importance spectrum plots and variable importances, to determine the relevance of genes.

**SignS** Molecular signatures and gene selection with survival and censored data.

- Implements a method that uses a combination of gene filtering, clustering and survival model building (FCMS), very similar to the one used in Dave et al. (2004).
- Honest assessment of model quality using cross-validation.
- Full details on models fitted and steps used, including detailed dendrograms, steps of variable selection, and correlation between signatures for FCMS.
- Comprehensive analysis of stability of solutions for both “best” number of genes, identity of selected genes, and signatures.
- Option to use validation data to obtain assessments of model quality.

**ADaCGH** Analysis of data from aCGH: calling gains and losses and estimating the number of copy changes in genomic DNA.

- Implements four methods that have been shown to perform well in previous studies (Willenbrock and Fridlyand 2005; Lai et al. 2005; Price et al. 2005): circular binary segmentation (Olshen et al. 2004), wavelet-based smoothing (Hsu et al. 2005), Price-Smith-Waterman SWARRAY (Price et al. 2005), and analysis of copy errors (the same method as implemented in CGH Explorer (Lingjaerde et al. 2005)).
- Diagnostic plots and overimposed plots to help determine suitability of methods and number of levels of gain/loss.

## Implementation

### Software and hardware infrastructure

Asterias runs on a computing and web-serving cluster with 30 nodes, each with two Xeon CPUs. This cluster uses Debian GNU/Linux as OS. We use Apache as web server, with web service load-balanced using Linux Virtual Server (LVS); because most computations are parallelized using MPI (see below), we use round-robin for webservice load-balancing. High-availability is achieved using redundancy in both LVS (two directors monitored with heartbeat) and storage (via a set of custom scripts). The database server for IDconverter and IDClight is MySQL.

All applications (except preP, DNMAD and Tnasas) are parallelized using the LAM/MPI implementation (http://www.lam-mpi.org) of MPI. Pomelo II is parallelized in C++, whereas the rest of the applications are parallelized in R using the library Rmpi (http://www.stats.uwo.ca/faculty/yu/Rmpi), and snow (http://www.stat.uiowa.edu/~luke/R/cluster/cluster.html) or papply (http://cran.rproject.org/src/contrib/Descriptions/papply.html). The MPI universe is created new for each run of each application, and the actual nodes to use in the MPI universe are determined at run-time after excluding possible non-responding nodes. This ensures that MPI can be used even if a node fails or is taken down for maintenance. When the parallelization does not involve all CPUs in the cluster, the CPUs used in the MPI universe are balanced: the configuration file for MPI depends on the master node of a run (and the master node is the one where the Apache process runs, which is balanced by LVS).

### Software

Computations are carried out using R and C/C++, either stand-alone or called from R. CGIs, data entry verification, MPI and cluster monitorization, and application counter and monitorization are written with Python, except for DNMAD (which use Perl). JavaScript is used for some of the dynamic output (collapsible trees, some clickable figures, and Ajax). Further details about design, implementation, and software and hardware organization, of interest mainly to developers, are provided at the Asterias project’s page (http://bioinformatics.org/asterias/wiki/Main/DevelopersDocumentation). As many other popular tools, we make extensive use of R and BioConductor packages, but many functions have been rewritten to allow for parallel computing. Full details on R/BioConductor packages used are provided on the help pages for each application.

### Testing

Testing that applications work as expected (see, e.g. Baxter et al. 2006) is an integral part of the development of Asterias. For most applications, a suite of tests, which use the FunkLoad tool (http://funkload.nuxeo.org/), is available from the the Asterias download page (http://bioinformatics.org/asterias/wiki/Main/DownloadPage) or from Launchpad (https://launchpad.net/projects/asterias). By using these tests we verify CGIs (including JavaScript), numerical output, the handling of error conditions and incorrectly formatted input files, and the setting up of MPI universes. For Pomelo II (currently the application that uses the most JavaScript and Ajax) we have also built tests (available from http://pomelo2.bioinfo.cnio.es/tests.html), using Selenium (http://www.openqa.org/selenium/), that verify that the application runs correctly under different operating systems and browsers.

### Users, application maturity, and bug-tracking

Asterias is a mature application suite, with a large number of users. Some of the applications that form part of Asterias have been running for almost three years (e.g. DNMAD, launched on October 2003) and the most recent applications (IDClight, preP) have been running since January 2006. The number of average daily uses (in the six-month period from 1-March-2006 to 1-September-2006) ranges from 5 per day for Pomelo II to 0.5 per day for SignS (Tnasas: 0.8; ADaCGH: 1.85; GeneSrF: 1; DNMAD: 3.88). For IDconverter the average daily uses are about 75 (IDClight uses are over 500, but each counted use involves a single identifier). Please note that the above are successful uses (i.e. only runs with validly formatted data sets are counted). Asterias now includes a bug-tracking and feature-requests page at http://bioinformatics.org/bugs/?group_id=630.

## User Interface

### Input

All applications use plain text files, with tab-separated columns for input. Missing values, in the applications that accept them, can be specified as either “NA” or by not filling the corresponding entry. The expression data files (**EDF**) (such as those returned by preP and used by all applications, except DNMAD and IDconverter) are formatted with genes in rows and patients or arrays in columns. The first column should be a column of identifiers, which can be of arbitrary length and include any character except tab (since tab is used for column separation). The array data can include a row with array/subject identifiers. It can also include an arbitrary number of comment lines (all lines with a “#” in the first column) anywhere in the file. Comment lines are a convenient way to record all transformations suffered by a file in DNMAD and preP.

DNMAD, IDconverter and IDClight have unique data entry requirements/flexibilities. For IDconverter the entry is a column of one or more identifiers. IDClight is designed for automated, programmable access, and accepts “data entries” via URL (see example in Table 1). DNMAD accepts either files in GPR format (http://www.moleculardevices.com/pages/software/gn_genepix_file_formats.html#gpr) as produced directly by many microarray scanners, or non-GPR files, if they have a specified set of columns (see DNMAD help).

The analysis applications need additional files: class information (e.g. Pomelo II), survival time and censored status (SignS, Pomelo II), and chromosome location information (ADaCGH) such as is returned by IDconverter. All these input files are also tab-separated, plain-text files. Further details are shown in Table 1.

### Output

**Figure 2.**
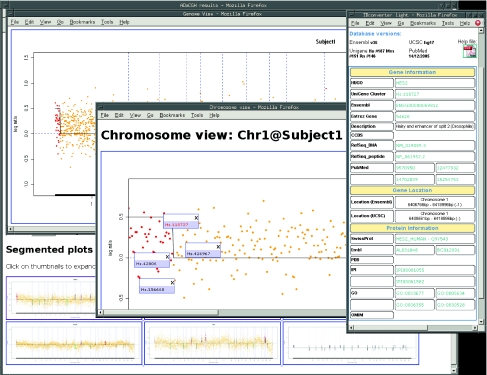
Output from ADaCGH. Partial output from ADaCGH showing: (Top) the bottom of the main output screen with the thumbnails for the segmented plots; (Bottom) Genome View for one of the arrays, obtained by clicking on the uppermost thumbnail in (Top); (Center) Chromosome View for the first chromosome (obtained by clicking on the region for the first chromosome in (Bottom)), with some data-points showing their ID; (Right) the results from IDClight obtained by clicking on the ID for one of the highlighted points in (Center).

**Figure 3.**
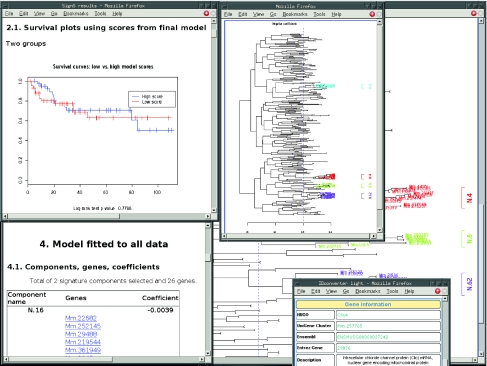
Output from SignS. Partial output from SignS showing: (Top left) Some model quality plots (survival plots from cross-validated prediction scores); (Bottom left) part of the output of the model fitted to the complete data set; note the clickable gene names; (Top center) the half-size dendrogram for the genes with negative coefficients, showing only clusters that fulfill the minimum requirements of correlation and size; (Right) same as (Top center), but using the double-size plot; (Bottom right) the results from clicking, on either (Top center) or (Right), on the cluster leave for Mm.257765.

A summary of the output of each application can be seen in Table 1. The complete output from each application, both tables and figures, can be saved to the user’s local file system, thus allowing for a detailed, complete record of the analysis. As shown in Figure 1, DNMAD and preP both produce output that can be sent to other applications of the suite, and SignS, ADaCGH, Pomelo II, Tnasas and GeneSrF provide clickable tables and figures that call IDClight. Examples of output are shown in Figures 2 and 3.

### Documentation and help

All applications have online help, most of them also include tutorials, detailed and commented examples, and sample data files, and Pomelo II also has additional tutorials as flash movies. Tutorials and examples are licensed under a Creative Commons license (http://www.creativecommons.org), thus allowing for redistribution and classroom use. In addition, courses on the use of our tools are taught occasionally.

## Future work

Our biggest development efforts are currently focused on two areas. We want to make Asterias easy to deploy at other places; to accomplish this, we are making available all of the source code as soon as it is ready for distribution (right now, all the testing code and some application’s code is available from http://bioinformatics.org/asterias/wiki/Main/DownloadPage or https://launchpad.net/projects/asterias), and we are using a general purpose web framework (Pylons: http://pylonshq.com) to ease distribution and installation. Releasing all of the code and making installation straightforward might draw other developers into Asterias. Another current effort focuses on increasing the use of parallelization and distributed computing to allow for faster responses and more efficient use of computational resources.

## Conclusions

Asterias is a freely-accessible suite of tools for the analysis of microarray data, both expression and aCGH, including normalization, missing data handling and imputation, differential gene expression, class prediction, survival analysis, and aCGH analysis. Asterias fully exploits its deployment in a cluster by using web-serving load-balancing and, more importantly, parallel computing for most of the computationally intensive tasks. Asterias also emphasizes sound and tested statistical approaches, provides careful analysis of the “multiplicity of solutions” problem, and integration of additional functional information.

## Figures and Tables

**Table 1.** Summary input and output for each application from the Asterias suite.

**Application**	**Input**	**Output**	
		**Tables and data sets**	**Figures**
DNMAD	GPR or custom format	Normalized log-ratios; A-values	Diagnostic plots.
preP	DNMAD output or EDF^1^	Post-processed EDF, summary statistics	
Pomelo II	preP output or EDF, class indicator, survival time and status	Differential expression statistics and p-values	Heatmap with gene dendrogram
Tnasas	preP output or EDF, class indicator	Error rates, selected genes, stability assessments	Cross-validated error rates vs. number of genes
GeneSrF	preP output or EDF, class indicator	OOB predictions, error rates, selected genes, stability assessments	OOB error vs. number of genes, OOB predictions, importance spectrum, selection probability plots.
SignS	preP output or EDF, survival time and status; optional validation files.	Single-gene statistics and p-values, CV predictions, model results and parameters, stability assessments	Survival plots, dendrograms, partial-likelihood plots.
ADaCGH	preP output or EDF and chromosomal location (e.g. from IDconverter)	Genes and segmented regions, summary statistics	Diagnostic plots, chromosome and genome segmented plots
IDconverter	identifiers	Mapped identifiers (gene, clone, protein), chromosomal location, PubMed abstracts, GO terms, pathways.	
IDClight	URL^2^	Same as IDconverter	

^1^ EDF: expression (or genomic) data file. See text for details.

^2^ For example, http://idclight.bioinfo.cnio.es/idclight.prog?idtype=ug&id=Hs.100890&org=Hs
